# Pulmonary Infiltrates in a Non-Cystic Fibrosis Bronchiectasis Patient: A Case Report

**DOI:** 10.3390/jcm14165914

**Published:** 2025-08-21

**Authors:** Francesco Rocco Bertuccio, Nicola Baio, Simone Montini, Valentina Ferroni, Vittorio Chino, Lucrezia Pisanu, Marianna Russo, Ilaria Giana, Elisabetta Gallo, Lorenzo Arlando, Klodjana Mucaj, Mitela Tafa, Maria Arminio, Emanuela De Stefano, Alessandro Cascina, Angelo Guido Corsico, Giulia Maria Stella, Valentina Conio

**Affiliations:** 1 Unit of Respiratory Disease, Cardiothoracic and Vascular Department, IRCCS Policlinico San Matteo, Viale Golgi 19, 27100 Pavia, Italy; francesco.bertuccio01@gmail.com (F.R.B.); simone.montini01@universitadipavia.it (S.M.); valentina.ferroni01@universitadipavia.it (V.F.); lucrezia.pisanu01@universitadipavia.it (L.P.); marianna.russo01@universitadipavia.it (M.R.); ilaria.giana01@universitadipavia.it (I.G.); elisabetta.gallo01@universitadipavia.it (E.G.); lorenzo.arlando01@universitadipavia.it (L.A.); klodjana.mukaj01@universitadipavia.it (K.M.); mitela.tafa01@universitadipavia.it (M.T.); maria.arminio01@universitadipavia.it (M.A.); emanuela.destefano01@universitadipavia.it (E.D.S.); a.cascina@smatteo.pv.it (A.C.); a.corsico@smatteo.pv.it (A.G.C.); 2 Department of Internal Medicine and Pharmacology, University of Pavia, 27100 Pavia, Italy; 3 Ospedale Maggiore, ASST Crema, 26013 Crema, Italy; nicola.baio01@universitadipavia.it; 4 Ospedale Pederzoli, Peschiera del Garda, 37121 Verona, Italy; vittorio.chino01@universitadipavia.it

**Keywords:** fungal infection, bronchiectasis, antifungal treatment

## Abstract

**Background:** *Scedosporium apiospermum* is a filamentous fungus increasingly recognized as an opportunistic pathogen in immunocompromised hosts, though rare infections in immunocompetent individuals with structural lung disease have been reported. Its diagnosis and management remain challenging due to non-specific clinical presentation and intrinsic resistance to multiple antifungal agents. **Case Presentation:** We report the case of a 66-year-old immunocompetent woman with idiopathic bilateral non-cystic fibrosis bronchiectasis, who presented with subacute cough and increased sputum production. Chest high-resolution CT revealed new subsolid and ground-glass infiltrates superimposed on stable bronchiectatic changes. Bronchoalveolar lavage (BAL) cultures isolated *S. apiospermum* as the sole pathogen. The patient was treated with oral voriconazole (200 mg BID) for 4 weeks, followed by a 4-week course of aerosolized amphotericin B. Clinical and radiological improvement was observed, and no relapse occurred during follow-up. **Discussion:** This case highlights the potential for *S. apiospermum* to cause clinically relevant pulmonary infection in structurally abnormal but immunocompetent lungs. Non-CF bronchiectasis may facilitate fungal colonization due to impaired mucociliary clearance and chronic mucus retention. Combined antifungal therapy involving systemic voriconazole and inhaled amphotericin B (though not yet standardized) was employed based on clinical rationale and the available literature, resulting in favorable outcomes. **Conclusions:** *S. apiospermum* pulmonary infection, although rare in immunocompetent hosts with bronchiectasis, should be considered in cases of new or persistent infiltrates. Early recognition and individualized antifungal strategies, including the potential role of inhaled agents, may improve clinical outcomes. This case reinforces the importance of multidisciplinary collaboration in the management of complex fungal infections in chronic airway disease.

## 1. Introduction

The *Scedosporium apiospermum* complex encompasses a group of filamentous fungi commonly found in environments, including soil, sewage systems, contaminated water sources, and agricultural settings. Based on the classification proposed by the European Confederation of Medical Mycology and the International Society for Human and Animal Mycology, this complex includes five phylogenetically related species: *S. apiospermum sensu stricto*, *Scedosporium boydii* (formerly *Pseudallescheria boydii*), *Scedosporium aurantiacum*, *Scedosporium dehoogii*, and *Scedosporium minutispora* [1,2].

Due to its wide range of associated clinical manifestations, the *S. apiospermum* complex is now recognized as one of the most prevalent mold pathogens capable of causing human infections. While it primarily acts as an opportunistic pathogen in immunocompromised individuals, cases have also been documented in immunocompetent hosts [3,4].

Clinical presentations can vary from superficial and localized infections of the skin and soft tissues, sometimes extending to deeper structures such as tendons, bones, and visceral organs, to severe disseminated infections. The lungs and lower extremities, particularly the feet, represent the most frequent sites of infection in immunocompetent individuals [5].

Pulmonary involvement by the *S. apiospermum* complex may manifest in several forms, including transient colonization of the respiratory tract, saprophytic colonization in structurally abnormal airways, hypersensitivity reactions resembling allergic bronchopulmonary mycosis, the formation of fungus balls (pseudallescherioma or scedosporioma), and rarely, invasive pseudallescheriasis or *Pseudallescheria*-associated pneumonia [6,7]. A well-defined pulmonary syndrome has been described in otherwise healthy individuals following near-drowning incidents in contaminated water [8]. However, lung infections caused by *S. apiospermum* in immunocompetent patients without such exposure history remain uncommon [3].

In vitro susceptibility studies have shown that *S. apiospermum* exhibits activity against several azole antifungal agents, including voriconazole, miconazole, albaconazole, posaconazole, and itraconazole. Among these, voriconazole is currently recommended as the first-line therapeutic agent for *S. apiospermum* infections [9,10].

In this report, we present a case of pulmonary infection due to *S. apiospermum* in an immunocompetent woman, which was ultimately resolved through combination of antifungals treatments.

## 2. Case Presentation

A woman in her sixth decade of life with a history of idiopathic bilateral non-cystic fibrosis bronchiectasis presented to our outpatient pulmonology clinic for follow-up complaining persistent cough and sputum production. She had a remote history of smoking (10 pack-years, quit in 1991), passive smoke exposure, and occupational history as an administrative clerk. Her comorbidities included gastroesophageal reflux disease and prior hysterectomy for uterine fibroids. The diagnosis of bronchiectasis was made about 10 years ago and was based on established HRCT criteria (including: (i) bronchial internal diameter greater than that of the accompanying pulmonary artery, (ii) lack of normal bronchial tapering, and (iii) visualization of bronchi within 1 cm of the pleural surface) with cylindrical bronchiectasis predominantly observed in the middle lobe and lingula, with additional segmental involvement of the left lower lobe.

In the past, she was hospitalized for episodes of hemoptysis, with subsequent isolation of *Haemophilus influenzae* and *Nocardia* spp. (treated with TMP/SMX, hospitalized for 2 weeks). Moreover, she was admitted for a lower respiratory tract infection caused by *Klebsiella oxytoca*, treated with intravenous meropenem. The patient was a retired office employee, without recent travel or relevant occupational/environmental exposures. She was not taking chronic medications at the time of presentation. The patient had not received systemic or inhaled corticosteroids, immunosuppressive medications, or other immunomodulatory agents prior to presentation.

## 3. Physical Examination Findings

During her outpatient follow-up she reported cough and increased sputum production; the patients made no complaints of fever, hemoptysis, or dyspnea.

At presentation, the patient appeared in no acute distress. Her vital signs were stable: no fever, heart rate 80 bpm, normal blood pressure and respiratory rate, and the oxygen saturation of 98% on room air. Lung auscultation revealed fine bilateral inspiratory crackles, without wheezes or rhonchi. No digital clubbing or peripheral edema was observed. Cardiac, abdominal, and neurologic examinations were unremarkable.

## 4. Diagnostic Studies

Blood tests, including complete blood count, liver and kidney function tests, electrolytes, and glucose, were all within normal limits. Inflammatory markers were not elevated (C-reactive protein, erythrocyte sedimentation rate). Previous HIV screening was negative together with Immunoglobulin levels (IgA, IgE, IgG, IgM) and alpha-1 antitrypsin (normal range). Serologic workup for connective tissue diseases was negative, including ANA, ANCA, and anti-SSA/SSB antibodies.

Pulmonary function tests showed normal dynamic lung volumes with a preserved diffusing capacity for carbon monoxide: FVC 93% of predicted; FEV1 100% of predicted; DLCO 88% of predicted.

**Figure 1:**
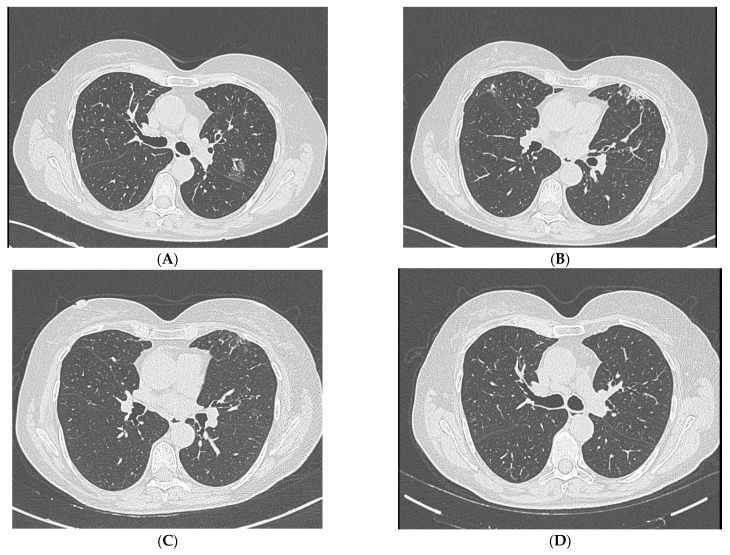
(**A**,**B**): High-resolution chest CT (HRCT) showing faint subsolid peribronchovascular infiltrate in the apical–posterior and anterior segment of the left upper lobe and new ground-glass opacity in the ventral segment of the right upper lobe, suggestive of inflammatory activity. (**C**,**D**): HRCT after antifungal therapy was performed showing partial resolution of inflammatory infiltrates.

Considering her past medical history, high-resolution chest CT (HRCT) was performed. It showed already known stable cylindrical bronchiectasis predominantly in the middle lobe and lingula, with mucous plugging and a new faint subsolid peribronchovascular infiltrate in the apical–posterior and anterior segment of the left upper lobe and new ground-glass opacities in the ventral segment of the left upper lobe, suggestive of inflammatory activity (Figure 1A,B).

A bronchoscopy with bronchoalveolar lavage was subsequently performed. Bronchoalveolar lavage (BAL) cultures yielded *Scedosporium apiospermum* as the only fungal isolate. A diagnosis of pulmonary infection by *Scedosporium apiospermum* in an immunocompetent patient with non-cystic fibrosis bronchiectasis was obtained. BAL fluid underwent fungal culture, bacterial culture, and mycobacterial culture.

Direct microscopic examination of BAL fluid revealed septate hyphae consistent with *Scedosporium* species. Identification was confirmed by MALDI-TOF mass spectrometry. Although fungal PCR and histopathology were not performed, the combination of compatible radiologic findings, isolation of *S. apiospermum* as the sole pathogen, and prompt clinical/radiological response to antifungal therapy supported the diagnosis of active pulmonary infection rather than colonization. PCR panels for respiratory viruses were negative. Serum galactomannan was negative, and β-D-glucan was not performed due to the expected low yield in localized infection in an immunocompetent host.

Pulmonary tuberculosis was excluded through absence of acid-fast bacilli on direct smear microscopy (negative Ziehl-Neelsen staining), negative Mycobacterium tuberculosis PCR on BAL samples as well as negative mycobacterial culture

Peripheral blood eosinophil count was within normal limits (100 cells/μL; reference range 0–500 cells/μL). Total serum IgE was 44 IU/mL (reference range < 100 IU/mL). The absence of central bronchiectasis, lack of migratory infiltrates on serial HRCT, and these laboratory findings rendered allergic bronchopulmonary mycosis (ABPM) due to *S. apiospermum* unlikely.

## 5. Clinical Course

Given the radiologic evolution and microbiologic isolation in a symptomatic patient, antifungal therapy with oral voriconazole (200 mg BID) was initiated after consultation with infectious disease specialists. Liver function tests were normal together with bilirubin levels and no significant drug–drug interactions were identified.

Follow-up HRCT was performed after antifungal therapy; it demonstrated persistent bilateral bronchiectasis, unchanged mucus plugging and partial resolution of inflammatory infiltrates (Figure 1C,D). Sputum culture was requested with no pathogens isolated. Moreover, patients referred showed improved symptoms and cough resolution. Voriconazole was discontinued after 4 weeks due to absence of clinical and radiological deterioration. Considering chronic lung alterations and impaired mucus clearance due to bronchiectasis, to avoid fungal colonization, additional local antifungal coverage was provided with a 4-week course of aerosolized amphotericin B (25 mg BID).

The patient is currently under regular follow-up after completion of antifungal therapy, with clinical evaluations performed at 1, 3, 6, and 12 months. Sputum cultures were not obtained due to absence of sputum production, and HRCT scans were performed at 6 and 18 months. No clinical relapse, radiologic progression, or fungal re-isolation occurred during follow-up.

## 6. Discussion

*Scedosporium apiospermum*, the anamorph of *Pseudallescheria boydii*, is an emerging opportunistic mold found in soil, polluted water, and decaying vegetation. While classically associated with infections in immunocompromised hosts its pathogenic potential in structurally abnormal but immunocompetent lungs is increasingly recognized [3,6].

Among immunocompetent cases, many (44% in the literature) had a prior history of pulmonary tuberculosis, supporting the hypothesis by Kantarcioglu et al. that prior TB infection may predispose to *S. apiospermum* pulmonary disease [11,12]. In immunocompromised populations, identified risk factors include lymphopenia, neutropenia, and hypoalbuminemia (<3 mg/dL). In contrast, in immunocompetent patients, risk factors tend to include antecedent trauma or surgery, with the respiratory tract being commonly affected sites [11]. The spectrum of pulmonary involvement includes transient colonization, saprobic airway colonization, fungus ball formation, and invasive pulmonary infection [3].

The clinical profile of pulmonary *S. apiospermum* infection typically includes non-specific respiratory symptoms: fever is the most frequently reported symptom, followed by cough, sputum production, hemoptysis, dyspnea, and pleuritic chest pain. Radiologically, findings often mimic those of other pulmonary infections such as aspergillosis. Imaging may reveal preexisting cavitary lesions containing fungal balls, nodular infiltrates (with or without cavitation), or more extensive bilateral infiltrates [13].

Definitive diagnosis requires microbiological, histopathological, or molecular approaches. *S. apiospermum* can be identified through direct microscopy, culture, and PCR-based methods for fungal DNA detection. Serologic testing (such as antigen detection by counter-immunoelectrophoresis or ELISA) may offer supportive evidence, although cross-reactivity with *Aspergillus* antigens limits its specificity and was not employed in this case [14,15,16].

Among immunocompetent patients (25 cases reported in the literature), the reported lethality rate from pulmonary *S. apiospermum* infection is 12.5%. These findings underscore the importance of individualized treatment strategies based on disease extent, host immune status, and response to antifungal therapy [6,14,17].

Importantly, our case demonstrates that *S. apiospermum* can cause clinically significant pulmonary disease in immunocompetent individuals even in the absence of prior trauma or surgical intervention, emphasizing the need for heightened clinical awareness and early evaluation in persistent or atypical respiratory infections.

Non-CF bronchiectasis represents a favorable environment for fungal colonization and infection due to impaired mucociliary clearance, chronic mucus stasis, and frequent antibiotic exposure (in Table 1 we summarize the main risk groups for *S. apiospermum* infection, the associated underlying conditions, and the typical sites of involvement). These alterations foster microbial dysbiosis and may promote overgrowth of filamentous fungi [18,19].

Voriconazole was selected based on its demonstrated in vitro efficacy against *S. apiospermum* and excellent pulmonary penetration. However, due to modest clinical improvement and persistent pulmonary infiltrates, adjunctive inhaled amphotericin B was administered.

The concept of combining systemic and inhaled antifungals, though not well-established for *Scedosporium* spp., draws support from studies in other molds, particularly *Aspergillus*. A study by Steinbach et al. (2015) demonstrated that combination therapy with voriconazole and amphotericin B in a murine model of invasive aspergillosis resulted in synergistic effects, reducing fungal burden and improving survival [20]. Furthermore, a study by Lackner et al. (2012) evaluating antifungal combinations against *Scedosporium* species reported partial synergism between triazoles and amphotericin B in select isolates [10].

Voriconazole remains the first-line agent due to its demonstrated in vitro activity and clinical efficacy against *S. apiospermum*, whereas systemic amphotericin B formulations are largely ineffective because of the pathogen’s intrinsic resistance. However, aerosolized amphotericin B, may achieve high concentrations within the airways and alveoli, potentially overcoming pharmacokinetic limitations and contributing to fungal burden reduction through topical antifungal effects [21,22,23].

Isolate-specific antifungal susceptibility testing was performed in our case, showing voriconazole sensitivity.

The rationale for this combination lies in the pharmacologic complementarity between agents: voriconazole provides deep tissue and systemic antifungal activity, while inhaled amphotericin B delivers high local concentrations that may exert direct fungicidal effects or disrupt biofilms, despite the organism’s systemic resistance profile. Moreover, aerosolized delivery may be particularly beneficial in anatomical settings where mucociliary clearance is impaired or drug penetration is suboptimal. This is especially relevant in bronchiectasis, where distorted airway architecture and chronic mucus retention create a niche for persistent fungal colonization and biofilm formation [24,25,26,27,28,29].

The decision to administer 4 weeks of systemic voriconazole followed by 4 weeks of inhaled amphotericin B was based on the patient’s immunocompetent status, localized disease without dissemination, rapid clinical improvement, and partial radiological resolution at 4 weeks. Sequential inhaled amphotericin B was chosen to maintain local antifungal effect while limiting systemic exposure. Similar shortened regimens have been reported in immunocompetent hosts with localized mold infections. It should be acknowledged that the evidence supporting the combination of voriconazole with amphotericin B in *S. apiospermum* is limited, and much of the rationale derives from studies in *Aspergillus* species and in vitro models. Robust clinical data for this specific pathogen are lacking.

Infections caused by this *Scedosporium apiospermum* should in non-immunocompromised patients with underlying bronchiectasis remain exceptionally rare (Table 2), with only isolated case reports documented in the literature (To our knowledge, only seven cases of pulmonary *S. apiospermum* infection in immunocompetent patients with non-CF bronchiectasis have been reported in the literature) [6,14,17,30,31,32,33]. The coexistence of this opportunistic mold with structurally abnormal airways represents an uncommon yet clinically significant intersection, often challenging in both diagnosis and therapeutic management. Currently, there are no universally accepted guidelines for the management of pulmonary *S. apiospermum* infections, resulting in highly variable therapeutic approaches. Given the pathogen’s intrinsic resistance patterns, potential for chronic colonization in structural lung disease, and the paucity of large-scale clinical trials, the development of evidence-based, consensus-driven guidelines is urgently needed to standardize diagnostic criteria, optimize antifungal regimens, and define the role of adjunctive inhaled therapies.

With the increasing prevalence of chronic respiratory diseases, greater survival of immunocompromised patients, and widespread use of broad-spectrum antibiotics and corticosteroids, the incidence of *S. apiospermum* infections may rise in the coming years. Enhanced environmental exposure due to climate change and urban wastewater distribution systems could also contribute to increased detection rates. Surveillance and mycological monitoring in high-risk populations will be key to anticipating this potential epidemiological shift.

This case illustrates that even in immunocompetent hosts, structural lung abnormalities such as non-CF bronchiectasis may predispose to opportunistic fungal infections. Impaired mucociliary clearance, chronic mucus stasis, and potential dysbiosis of the airway microbiota create a permissive environment for fungal growth and progression from colonization to true infection. Clinicians should maintain a high index of suspicion for filamentous fungi, including *S. apiospermum*, in patients with bronchiectasis who develop new or persistent pulmonary infiltrates.

Compared with the seven previously published cases of *S. apiospermum* pulmonary infection in immunocompetent, non-CF bronchiectasis patients, our case is distinguished by the sequential use of systemic voriconazole followed by inhaled amphotericin B, with documented sustained remission over a substained follow-up period. This therapeutic approach and outcome add valuable evidence to the limited literature on optimal management in such patients. Further research is warranted to define optimal therapeutic regimens.

## 7. Conclusions

Reports of *S. apiospermum* infection in immunocompetent, non-CF bronchiectasis patients remain rare. Documenting such occurrences expands our understanding of fungal epidemiology in chronic lung disease. There is no standardized treatment guideline for *Scedosporium* pulmonary infections. Our use of sequential systemic and inhaled antifungals illustrates a pragmatic, individualized approach. Effective management relied on close collaboration between pulmonologists, infectious disease specialists, and radiologists, emphasizing the importance of team-based care.

## Figures and Tables

**Table 1:** Summary of risk groups for *S. apiospermium* infections.

Risk Group	Underlying Conditions/Risk Factors	Typical Infection Site(s)
Immunocompromised patients	Hematologic malignancies, solid organ/stem cell transplantation, prolonged neutropenia, corticosteroid therapy	Lungs, CNS, disseminated
Immunocompetent with structural lung disease	Non-CF bronchiectasis, prior pulmonary tuberculosis, COPD, cystic lung disease	Lungs
Post-trauma or surgery	Penetrating injuries, orthopedic implants, post-surgical wounds	Skin, soft tissue, bone
Near-drowning victims	Aspiration of contaminated water	Lungs, CNS
Patients with chronic sinusitis or otitis	Anatomical alterations, recurrent bacterial infections	Paranasal sinuses, ear
Environmental exposure without apparent host factors	Soil or sewage contact in endemic areas	Variable

**Table 2:** Summary of non-immunocompromised patients with bronchiectasis and pulmonary *Scedosporium apiospermum* infection.

Reference (Year)	Age/Sex	Imaging Findings	Symptoms	Diagnostic Method	Treatment	Outcome
**Liu et al. (2020)** [6]	44/F	Cavitary lesion and consolidation in the left upper lobe	Hemoptysis, cough, weight loss, anorexia	BAL culture	Voriconazole 200 mg BID × 8 wk → lobectomy → VRC × 6 mo	Complete resolution
**Hassan et al. (2010)** [30]	26/M	Pneumothorax and cavitary mycetoma	Cough, expectoration, fever, spontaneous pneumothorax, fungal empyema	BAL culture	Voriconazole and surgical resection	Recovery
**Mir et al. (2021)** [17]	83/F	Persistent infiltrates and tree in bud	shortness of breath, cough with blood-tinged sputum, and fatigue for the past several months	Sputum culture	Voriconazole 6 mg/kg intravenously twice a day for the first 24 h, followed by 4 mg/kg twice-daily dosing. At the time of discharge to home, the patient was kept on oral voriconazole 200 mg twice daily for 6 months	Clinical improvement, radiologic resolution
**Cruz et al. (2015)** [31]	67/F	Cavitary lesion with adjacent bronchiectasis	Persistent cough with bronchorrhea, hemoptisis, fever and general condition impairment	BAL culture	Itraconazole (failed) → Voriconazole × 16 wk	Favorable response
**Jimeno et al. (2020)** [33]	74/F	RUL cavitary lesion	Asymptomatic	BAL culture	Voriconazole × 3 wk	Improvement, later deterioration due to other causes
**Durant et al. (2011)** [32]	61/F	Mycetoma in right upper lobe;	Hemoptysis	Sputum and BAL Culture	Voriconazole × 3 months	Hemoptysis resolved, stable imaging

## Data Availability

The data that support the findings of this study are available on request from the corresponding author.
